# Identification of Sequences Encoding *Symbiodinium minutum* Mitochondrial Proteins

**DOI:** 10.1093/gbe/evw002

**Published:** 2016-01-21

**Authors:** Erin R. Butterfield, Christopher J. Howe, R. Ellen R. Nisbet

**Affiliations:** ^1^School of Pharmacy and Medical Sciences, Sansom Institute for Health Research, University of South Australia, North Terrace, Adelaide, SA, Australia; ^2^Department of Biochemistry, University of Cambridge, Cambridge, Cambridgeshire, United Kingdom

**Keywords:** metabolism, mitochondria, alveolate, chloroplast, dinoflagellate

## Abstract

The dinoflagellates are an extremely diverse group of algae closely related to the Apicomplexa and the ciliates. Much work has previously been undertaken to determine the presence of various biochemical pathways within dinoflagellate mitochondria. However, these studies were unable to identify several key transcripts including those encoding proteins involved in the pyruvate dehydrogenase complex, iron–sulfur cluster biosynthesis, and protein import. Here, we analyze the draft nuclear genome of the dinoflagellate *Symbiodinium minutum*, as well as RNAseq data to identify nuclear genes encoding mitochondrial proteins. The results confirm the presence of a complete tricarboxylic acid cycle in the dinoflagellates. Results also demonstrate the difficulties in using the genome sequence for the identification of genes due to the large number of introns, but show that it is highly useful for the determination of gene duplication events.

Dinoflagellates are a very diverse group of eukaryotic organisms. Many species are photosynthetic, and some are important coral symbionts. Other species are nonphotosynthetic, and can cause toxic algal blooms and paralytic shellfish poisoning. Dinoflagellates are a sister group to the Apicomplexa, a group of primarily intracellular parasites which include the malaria agent *Plasmodium*. The majority of the Apicomplexa have lost the ability to carry out photosynthesis, yet retain a remnant plastid. Thus, dinoflagellates provide a unique opportunity to examine the metabolic changes required in the conversion from a photosynthetic to a parasitic life style.

Little is known about dinoflagellate biochemistry. Until recently there have been very limited DNA sequence data available. In 2013, three extensive studies examined all available dinoflagellate expressed sequence tag (EST) and RNAseq data in order to identify and annotate biochemical pathways present within dinoflagellate species. All three studies were unable to identify sequences encoding many key proteins involved in a variety of biochemical pathways (Butterfield et al. 2013; Danne et al. 2013; Wisecaver et al. 2013). These included the following: NAD^+^ (nicotinamide adenine nucleotide) isocitrate dehydrogenase (involved in the tricarboxylic acid [TCA] cycle), complex I of the mitochondrial electron transport chain (ETC), and Isd11 (involved in the iron–sulfur cluster biosynthesis pathway). Furthermore, a typical pyruvate dehydrogenase complex (PDH) appeared to be absent, and it was suggested that it had been replaced with either a pyruvate:ferredoxin oxidoreductase (PFO), the branched chain α-ketoacid dehydrogenase complex, or a *Corynebacterium-*style PDH (Butterfield et al. 2013; Danne et al. 2013; Wisecaver et al. 2013). One of the surprising findings was the identification of very few proteins involved in mitochondrial protein import, suggesting that the import apparatus is minimal, or that it is very divergent (Butterfield et al. 2013).

The first dinoflagellate nuclear genome sequence reported was for *Symbiodinium minutum* (Shoguchi et al. 2013). The *S. minutum* nuclear genome is approximately 1,500 Mbp in size and is extremely intron rich, which made sequencing and annotating the genome a significant achievement. RNAseq data were also released for *S. minutum* (Shoguchi et al. 2013). We therefore analyzed the nuclear genome and related transcriptome data to identify genes involved in key metabolic pathways which had not been identified in the previous studies.

## Multiple Introns Prevent Gene Identification

One hundred and eleven proteins (primarily mitochondrial) not previously identified in the dinoflagellates were searched against the *S. minutum* nuclear genome using the BLAST (Basic Local Alignment Search Tool) algorithm (supplementary table S1, Supplementary Material online). Queries included protein sequences from a range of organisms and 26 nucleotide sequences (both as nucleotide and translated) from several dinoflagellate species including *Perkinsus* and/or *Oxyrrhis* (nonphotosynthetic early-branching dinoflagellate species) and *Chromera* (a photosynthetic apicomplexan). Five genes encoding putative mitochondrially targeted proteins were identified in the BLAST searches: Tim17 (protein import), adrenodoxin NADP + oxidoreductase, cytochrome *c* or *c*1-type heme lyase, NAD^+^ isocitrate dehydrogenase, and an aminomethyltransferase (table 1).

**Table 1:** Genes Identified by BLAST as Present in the *Symbiodinium minutum* Nuclear Genome

Protein Name	Pathway	Location
NAD + isocitrate dehydrogenase	TCA cycle	scaffold4965.1:12909-13436,13559-13693,13786-13983
Cytochrome *c*-type heme lyase	ETC assembly	scaffold4113.1:17855-17682
NADPH adrenodoxin oxidoreductase	Fe–S cluster biosynthesis/ETC assembly	scaffold2449.1:42386-43780
Aminomethyltransferase	Unclear	scaffold55.1:19483-19082
Tim17	Protein import	scaffold1137.1:25614-25411,25297-25226,25163-25110
Ferredoxin NADP reductase (chloroplast) (cpFNR)	Photosynthesis	scaffold1625.1:140107-139538,scaffold7056.1:11328-12305,scaffold996.1:74923-75258,75279-75019,77412-77011,scaffold1066.1:78934-79236,scaffold1066.1:75430-75732,scaffold303.1:30938-31327,scaffold5347.1:2431-2550,3378-3323,3572-3439,scaffold4627.1:33578-33706

The number of genes identified was considerably lower than expected. We therefore altered the gap penalties to determine whether this would increase detection of genes present in the *S. minutum* nuclear genome. Previously successful query sequences or dinoflagellate ESTs encoding NAD^+^ isocitrate dehydrogenase, Tim17, and Tom40 (*Tetrahymena thermophila* EAR84154, *Plasmodium falciparum* AAN36941, and *Alexandrium minutum* GW810016, respectively) were used to search the genome with all seven different gap penalties available on the online version of BLAST. Altering the gap penalties did not alter the results obtained for NAD^+^ isocitrate dehydrogenase or Tim17, with the number of sequences which met the *E*-value threshold remaining the same.

For Tom40, the top hit contained four sequences. However, all were identified with *E* values greater than the threshold of 1 × 10^−10^ (i.e., 1 × 10^−6^, 0.001, 0.58, and 6.0). Two of these sequences were present for all gap penalties tested. These four sequences are located within 4 kb on scaffold 344.1 (supplementary table S2, Supplementary Material online). Although results for the first sequence when analyzed by Blastx suggested that it encoded Tom40 or a eukaryotic porin domain-containing protein, the *E* value obtained in the blastx search (2 × 10^−4^) was well above the threshold*.* However, when the EST transcript which mapped to this region (Shoguchi et al. 2013) was analyzed by blastx, results indicated that the transcript most likely encoded Tom40 or a eukaryotic porin domain-containing protein (with an *E* value well below the threshold). Therefore, although each of the original sequences identified did encode regions of Tom40, the BLAST results were well above the threshold level, even with altered gap penalties and remained undetected in our search. This is likely to be due to the *Tom40* gene being represented by many small exons separated by large introns, with a 1.2-kb gene spanning 14 kb of genomic sequence.

The presence of multiple introns may therefore explain why so few genes were identified, as shown by our results of the altered gap penalties test. The mean length of a gene is approximately 12 kb, including approximately 19 introns (of mean length 499 bp), although some genes have up to 256 introns (Shoguchi et al. 2013). Thus, the mean length of a single exon is approximately 100 bp, although many must be considerably shorter (Shoguchi et al. 2013). Coupled with sequence divergence, this extreme fragmentation decreases the ability of algorithms based on sequence alignment to recognize gene sequences, due to the very short exon size. To determine whether this was indeed the case, a positive control experiment was performed. Previously identified *Symbiodinium* sequences encoding mitochondrial proteins (Butterfield et al. 2013) were used as queries to search the *S. minutum* nuclear genome and transcriptome as either nucleotide sequences (blastn) or as protein sequences (tblastn) (details of sequences used are in supplementary table S3, Supplementary Material online). To determine whether the high number of introns may be influencing the results, a third search was performed where the translated ESTs were searched against the *S. minutum* nuclear genome using a max intron length setting of 499 bp (average intron length) (Shoguchi et al. 2013). Of the 25 nucleotide sequences, only 7 sequences (28%) were identified in the *S. minutum* nuclear genome using blastn. This increased to 13 of the 25 (52%) sequences when using tblastn (default settings) (supplementary table S4, Supplementary Material online). Using an increased maximum intron size further increased this to 16 of the 25 sequences (64%). However, the best results were obtained when searching the transcriptome using tblastn (22/25, 88%). For full results, see supplementary table S4, Supplementary Material online. In all cases, a threshold of 1 × 10^−10^ was applied. In some cases, a gene was identified multiple times, in different regions of the genome, indicating gene duplication events. Interestingly, duplicated genes were not always identified by both blastn and tblastn, with individual searches identifying different regions of the genome.

Together, these results suggest that the high number of introns in the dinoflagellate genome prevents the identification of genes using the BLAST algorithm, even when using very relaxed gap penalties and increased intron size settings. These results also suggest that the transcriptome is more reliable for determination of gene presence within *S. minutum* than the nuclear genome sequence.

## Identification of Additional Genes Using Transcriptome Data

As the positive control experiment indicated a greater detection of *S. minutum* encoded genes using transcriptomic data, the initial search for dinoflagellate mitochondrial encoded genes was repeated using the transcriptome. Transcripts for an additional 22 genes were identified (as shown in table 2).

**Table 2:** Transcripts Identified Using BLAST to Search the *Symbiodinium minutum* Transcriptome

Protein Name	Pathway	Transcripts	Present	Notes
Glucokinase	Glycolysis	symbB1.comp15755_c0_seq1, symbB1.comp22099_c0_seq1, symbB1.comp17604_c1_seq1, symbB1.EST_k37c20_20518, symbB1.EST_k37c20_28671, symbB1.EST_k37c20_9232	Yes	
Hexokinase	Glycolysis	symbB1.comp35096_c0_seq1	Possible?	
PFO/PNO	PDH	symbB1.EST_k37c20_3908, symbB1.EST_k37c20_3906, symbB1.EST_k37c20_3904, symbB1.EST_k37c20_3903, symbB1.EST_k37c20_3910, symbB1.EST_k37c20_3907, symbB1.EST_k37c20_3905, symbB1.EST_k37c20_3909	Yes	
NAD + isocitrate dehydrogenase	TCA cycle	symbB1.comp29416_c0_seq1, symbB1.EST_k37c20_25209	Yes	
Ferredoxin-NADP reductase	ETC assembly/Fe–S cluster biosynthesis	symbB1.EST_k37c20_33041, symbB1.EST_k37c20_17946, symbB1.EST_k37c20_23228, symbB1.EST_k37c20_11157, symbB1.EST_k37c20_11158, symbB1.EST_k37c20_11159, symbB1.EST_k37c20_149, symbB1.EST_k37c20_5213, symbB1.EST_k37c20_5214, symbB1.comp31035_c0_seq1	Yes	
Cytochrome *c*-type heme lyase	ETC assembly	symbB1.comp14560_c0_seq1, symbB1.comp5239_c0_seq1, symbB1.EST_k37c20_44663, symbB1.EST_k37c20_4983,	Yes	
CcsA/CcsB	ETC assembly	symbB1.comp705_c0_seq1	Possible?	Involved in chloroplast cytochrome *c* assembly pathways
Ccb3	ETC assembly	symbB1.EST_k37c20_7170	Unclear	Involved in chloroplast cytochrome *c* assembly pathways
Isd11	Fe-S cluster biosynthesis	symbB1.comp12486_c0_seq1	Probable	
Jac1	Fe-S cluster biosynthesis	symbB1.comp43118_c0_seq1, symbB1.EST_k37c20_38150	Yes	
Isa1	Fe-S cluster biosynthesis	symbB1.comp10688_c0_seq1, symbB1.EST_k37c20_59904	Yes	
Iba57	Fe-S cluster biosynthesis	symbB1.comp43847_c0_seq1, symbB1.EST_k37c20_46197	Possible?	
Glutamyl-tRNA reductase	Heme biosynthesis	symbB1.comp6620_c0_seq1, symbB1.comp12025_c0_seq1, symbB1.EST_k37c20_14582, symbB1.EST_k37c20_9955	Yes	Likely chloroplast targeted
Gun4	Tetrapyrrole biosynthesis	symbB1.comp2323_c0_seq1, symbB1.EST_k37c20_13544^a^	Yes	Likely chloroplast targeted
Magnesium chelatase subunit D	Tetrapyrrole biosynthesis	symbB1.comp5397_c0_seq1, symbB1.comp8938_c0_seq1, symbB1.EST_k37c20_13445^a^, symbB1.EST_k37c20_16659, symbB1.EST_k37c20_26080	Yes	Likely chloroplast targeted
DHFS/FPGS	Folate biosynthesis	symbB1.EST_k37c20_17505, symbB1.comp50374_c0_seq1	Yes	
Holocarboxylase synthetase	Biotin biosynthesis	symbB1.EST_k37c20_34625	Yes	
Tom40	Protein import	symbB1.comp10772_c0_seq1, symbB1.EST_k37c20_2453	Yes	
Tom70	Protein import	symbB1.EST_k37c20_13157, symbB1.EST_k37c20_42022, symbB1.comp40750_c0_seq1	Possible?	
Tim50	Protein import	symbB1.comp8394_c0_seq1, symbB1.comp12755_c0_seq1^a^, symbB1.comp28424_c0_seq1^a^, symbB1.comp24184_c0_seq1^a^, symbB1.comp5472_c0_seq1^a^, symbB1.EST_k37c20_15826^a^, symbB1.EST_k37c20_25066^a^, symbB1.EST_k37c20_25650^a^, symbB1.EST_k37c20_28256, symbB1.EST_k37c20_38959^a^	Yes	
Tim17	Protein import	symbB1.comp9012_c0_seq1, symbB1.comp38293_c0_seq1, symbB1.EST_k37c20_21411, symbB1.EST_k37c20_47221	Yes	
Tim14	Protein import	symbB1.comp14121_c0_seq1, symbB1.EST_k37c20_6257	Yes	
Tim9/Tim10	Protein import	symbB1.comp13846_c0_seq1symbB1.EST_k37c20_43233	Yes	
Tim10/Tim13	Protein import	symbB1.comp12759_c0_seq1, symbB1.comp24056_c0_seq1	Yes	
Tim10		symbB1.EST_k37c20_29860	Yes	
Inner membrane protease 1	Protein import	symbB1.comp32073_c0_seq1	Possible?	
Inner membrane protease 2	Protein import	symbB1.comp29930_c0_seq1, symbB1.EST_k37c20_32150^a^, symbB1.EST_k37c20_35068^a^	Possible?	
cpFNR	Photosynthesis	symbB1.comp45_c0_seq1, symbB1.comp1697_c0_seq2, symbB1.comp60704_c0_seq1, symbB1.EST_k37c20_4294, symbB1.EST_k37c20_46801	Yes	Likely chloroplast targeted

^a^Indicates gene identified in genome search only.

A single transcript was identified as a possible hexokinase; however, blastx analysis of the transcript showed the only hexokinase match to be from *Pfiesteria piscicida* (ACU45010.1). blastp of the *Pf. piscicida* protein sequence suggests that it may have been misidentified, as it does not detect any known hexokinase sequences. A transcript encoding a glucokinase was identified, suggesting that *S. minutum* like *Perkinsus marinus* and the ciliates likely uses a glucokinase rather than a hexokinase for the conversion of glucose to glucose-6-phosphate (Smith et al. 2007; Butterfield et al 2013).

Transcripts were identified encoding a PFO or pyruvate:NADPH oxidoreductase (PNO). Transcripts for these genes had previously been identified in *Pe. marinus* and *Alexandrium tamarense* (Butterfield et al. 2013; Wisecaver et al. 2013). No transcript was identified for the bacterial-type E1 subunit of PDH. This is in contrast to Butterfield et al. (2013) and Wisecaver et al. (2013) who identified sequences encoding a bacterial-type subunit in *Amphidinium carterae* and *Al. tamarense*, respectively (Butterfield et al. 2013; Wisecaver et al. 2013). These results suggest that biochemical analyses will be required to fully understand PDH evolution and pyruvate metabolism within the dinoflagellates.

The identification of a sequence encoding an NAD^+^-linked isocitrate dehydrogenase shows that there is a complete TCA cycle present within the dinoflagellates. This confirms the *Pe. marinus* metabolomic studies conducted by Danne et al. (2013) which identified key TCA cycle metabolites. Transcripts encoding NAD^+^ isocitrate dehydrogenase were not identified in previous EST data analyses, most likely due to transcripts being at very low abundance (Butterfield et al. 2013; Danne et al. 2013; Wisecaver et al. 2013).

The attachment of heme to apocytochrome *c* for the mitochondrion can be carried out by one of the three pathways: System I, System III, or System V. Each species contains just one system (Allen et al. 2008; Allen 2011). Analyses of the cytochrome *c*-type heme lyase gene showed it to encode either a *c*- or *c*1-type lyase, consistent with the *c*- or *c*1-type heme lyase previously identified within *P. marinus* (Butterfield et al. 2013). Together these results confirm that the dinoflagellates, including *Pe. marinus*, contain the System III cytochrome *c* biogenesis pathway (Allen et al. 2008; Allen 2011). Although the apicomplexan *Plasmodium* also contains a System III cytochrome *c* biogenesis pathway, it encodes two heme lyases rather than one (van Dooren et al. 2006). The transcripts identified in *S. minutum* map to three different scaffolds (Shoguchi et al. 2013) suggesting that there are at least three cytochrome *c*- or *c*1-type heme lyases encoded on the *S. minutum* nuclear genome. Transcripts were also identified for genes involved in the System II and System IV pathways utilized within the chloroplast (Allen et al. 2011).

There are three pathways for the synthesis of iron–sulfur clusters, essential protein cofactors. The ISC pathway (iron–sulfur cluster) is found in the mitochondrion (Seeber 2002; Nývltová et al. 2013). Previous studies of dinoflagellate transcript data had failed to identify numerous sequences encoding essential proteins in the ISC pathway. These included Isd11 (involved in the release of sulfides from cysteine), Jac1 (a cochaperone), Iba57, Isa1 (both essential for the transfer of iron–sulfur clusters to apoproteins), and adrenodoxin NADP + oxidoreductase (Butterfield et al. 2013; Danne et al. 2013). Although we were unable to identify genes encoding Isd11, Jac1, or Isa1 in the *S. minutum* nuclear genome, transcripts were identified for Jac1, Isa1, and a probable transcript was identified for Isd11. We were also able to identify a gene encoding an adrenodoxin NADP oxidoreductase and an aminomethyltransferase, which could correspond to Iba57 (genome: 8 × 10^−15^, 33% identity to *Homo sapiens*; transcriptome: 1 × 10^−24^, 36% identity to *H. sapiens*). These results are highly supportive of the presence of a complete ISC pathway within the dinoflagellates. The identification of adrenodoxin NADP + oxidoreductase is an important step in identifying the genes encoding proteins involved in both the iron–sulfur cluster biosynthesis and ETC assembly pathways (Barros et al. 2002; Lill and Mühlenhoff 2005).

Several proteins involved with protein import into the mitochondrion had not been previously identified within the dinoflagellates (Butterfield et al. 2013). A search of the *S. minutum* nuclear genome was able to identify only one further component of the protein import apparatus, Tim17. However, analysis of the transcriptome was also able to identify Tom40, Tim50, Tim14, and Tim10 or Tim13. Additionally, transcripts were identified which may encode Tom70, Tim9, and inner membrane protease 1 and 2. The identification of these additional transcripts suggests that dinoflagellate protein import is a more complex process than initial results suggested (Butterfield et al. 2013) although still appears to be highly reduced, similar to that present in Microsporidia, *Plasmodium*, and *Cryptosporidium* (van Dooren et al. 2006; Heinz and Lithgow 2013).

EST analyses have previously identified a transcript encoding glutamyl-tRNA reductase in *Lingulodinium* (Butterfield et al. 2013; Danne et al. 2013) which had been suggested to be contaminated due to the Guanine-Cytosine content and amino acid sequence (Butterfield et al. 2013**)**. We were unable to identify a sequence encoding glutamyl-tRNA reductase on the *S. minutum* nuclear genome; however, we were able to identify a transcript. This may suggest that the transcript identified by both Danne et al. (2013) and Butterfield et al. (2013) in the *Lingulodinium* database may not be contamination. As no sequence was identified in the genome or the transcriptome for the previously identified potential contaminant transcript encoding YaeT (an alternative to the SAM complex involved in protein import), it supports the suggestion of contamination in the *Oxyrrhis marina* library (Butterfield et al. 2013).

Transcripts were also identified for various cofactor synthesis pathways including folate biosynthesis and biotin biosynthesis. *Symbiodinium minutum* contains sequences encoding a dual dihydrofolate synthase–folylpolyglutamate synthase (DHFS-FPGS) like that present within *Pl. falciparum* (Salcedo et al. 2001) and in *T. thermophila* (XP_001010006.3) which may suggest that the alveolate ancestor contained the dual version of the enzyme. A transcript was also identified for holocarboxylase synthetase involved in the biotin biosynthesis pathway, therefore the only protein which remains unidentified in this pathway is dethiobiotin synthase (Butterfield et al. 2013). The absence of this is not surprising as this gene has not been identified in any algal species or *Arabidopsis thaliana* (Croft et al. 2006). Recently, sequences encoding a protein of dual function (diaminopelargonic acid aminotransferase/dethiobiotin synthetase [Bio3-Bio1]) were identified in *Ar. thaliana* and some algal species (Muralla et al. 2008; Cobessi et al. 2012); however, no gene or transcript for this was identified in *S. minutum*. It is likely that the dethiobiotin synthesis reaction is catalyzed by an unknown mechanism (Croft et al. 2006).

## Gene Duplications Are Common

Eight of the 25 genes identified from the positive control experiment against the genome sequence (i.e., *Symbiodinium* sequences which had been previously identified through analysis of EST data by Butterfield et al. 2013) returned BLAST results with more than one location on the genome scaffolds. This is suggestive of gene duplication. Analysis of mapped RNA transcripts (Shoguchi et al. 2013) suggested that under the conditions in which the library was made, not all copies of duplicated genes may be transcribed. For example, two copies of the cytochrome *c* gene were identified. The two genes are arranged in tandem. The first gene encodes a protein with a longer N-terminal region than the product of the second gene, there is a single nucleotide substitution between the genes, and each gene has a different 3′ UTR (untranslated region). An alignment is shown in supplementary alignment S1, Supplementary Material online. All corresponding *S. minutum* RNA transcripts encode a protein with the longer N-terminal region, as well as having the nucleotide substitution and 3′ UTR corresponding to the first cytochrome *c* gene. There are no transcripts corresponding to the second gene. This suggests that the second gene may not be transcriptionally active, at least under the conditions where the RNA transcripts were obtained. The significant sequence similarity between the two copies of the cytochrome *c* gene would suggest that this gene duplication is recent.

Apicomplexa and the early branching dinoflagellate *Perkinsus* all contain sequences encoding mitochondrial ferredoxin NADP reductase (FNR) (adrenodoxin NADP + oxidoreductase) (Lei et al. 2010). However, initial searches using the dinoflagellate EST libraries were only able to identify a putative FNR homolog which showed more similarity to the chloroplast isoform (cpFNR). During the search for the mitochondrial isoform on the *S. minutum* nuclear genome (table 1), eight genes encoding putative cpFNRs were also identified. An analysis of the transcripts mapped to these regions suggested that all eight genes are transcribed, at least partially. Alignments of the translated sequences showed that the genes fall within three groups, suggesting that there were three original cpFNR genes which have each undergone gene duplication (supplementary alignment S2, Supplementary Material online). Interestingly, the transcript aligned to one of the likely cpFNRs (4627.1) appears to include two frameshift mutations, due to the insertion of a single nucleotide at two different sites in the RNA. It is unclear whether this is caused by posttranscriptional editing or is a result of sequencing errors. No other instances of possible editing were detected, suggesting that sequencing error is more likely.

## Conclusion

Despite extensive searches of the *S. minutum* nuclear genome, we were able to identify only a further five genes encoding mitochondrial proteins. This increased to 27 when transcriptomic data were used. There are multiple reasons for the extremely low success rate in gene identification using the *S. minutum* nuclear genome sequence. The most likely reason, supported by the results of the positive control experiment, is the very high level of introns in genes, and the fact that exon size is small. Second, the genome remains in draft form with numerous scaffolds and contigs, one of which is known to be bacterial contamination (Shoguchi et al. 2013). Although the *S. minutum* nuclear genome has been estimated to be approximately 1,500 Mbp, at present only 616 Mbp (41%) has been sequenced and released (Shoguchi et al. 2013). Although the sequenced portion of the genome has been suggested to be the euchromatin-like region of the *S. minutum* genome, as the majority of the transcripts can be mapped, it is possible that the genes that we failed to identify in this study are located in the 59% of unsequenced genome. Finally, some genes may be truly absent from *S. minutum*.

Despite the difficulties in using the nuclear genome for the identification of the genes present within *S. minutum*, the genome sequence has been shown to be very useful for the identification of gene duplication events. Furthermore, the identification of 27 additional genes increases our knowledge of dinoflagellate biochemical pathways, including those shared with the Apicomplexa. The further curation of the *S. minutum* nuclear genome sequence will enable the continued characterization of shared pathways, increasing our understanding of how photosynthesis is lost. However, for now, as indicated by our positive control experiment, it may be better to rely on the extensive EST databases for the inference of gene content within the dinoflagellate algae.

## Methods

The *S. minutum* nuclear genome sequence and transcriptome were downloaded from the OIST Marine Genomics Unit website http://marinegenomics.oist.jp/symb/viewer/download?project_id=21 (last accessed September 30, 2013) (Marine Genomics Unit 2011). The original query sequences from Butterfield et al. (2013) and further query sequences were obtained from the National Center for Biotechnology Information and used to search the *S. minutum* nuclear genome. Obtained nucleotide query sequences were translated using the ExPASy translate tool (http://web.expasy.org/translate/, Swiss Institute of Bioinformatics). Nucleotide or protein input sequences were analyzed using either BLASTN or TBLASTN against the *S. minutum* nuclear genome and the transcriptome (Altschul et al. 1997, 1990). The genome location (scaffold number and region) or transcript identification were recorded for sequences that returned an *E* value of less than or equal to 1 × 10^−10^. Hits and corresponding RNA transcripts overlaying the region identified on the genome (Shoguchi et al. 2013) were analyzed with blastx to confirm identification. *Symbiodinium minutum* listed transcripts are from two different libraries (Trinity and Velvet/Oasis assemblies). Transcripts from the Trinity library were analyzed. If no transcript was found in the Trinity library, then the Velvet/Oasis library was used instead.

## Supplementary Material

Supplementary tables S1–S4 are available at *Genome Biology and Evolution* online (http://www.gbe.oxfordjournals.org/)

Supplementary Data
