# Supplementary material for: Identification of Sequences Encoding Symbiodinium minutum Mitochondrial Proteins
Source: Genome Biol Evol. 2016 Jan 21;8(2):439–45. doi: 10.1093/gbe/evw002 (PMC4779609; doi:10.1093/gbe/evw002)
Supplement: Supplementary Data [file supp_8_2_439__index.html]

Identification of Sequences Encoding Symbiodinium minutum Mitochondrial Proteins — Supplementary Data 

# Identification of Sequences Encoding *Symbiodinium minutum* Mitochondrial Proteins

## Supplementary Data

files

- Supplementary Data - docx file
- Supplementary Data - xlsx file
