# Supplementary material for: Identification of Sequences Encoding Symbiodinium minutum Mitochondrial Proteins
Source: Genome Biol Evol. 2016 Jan 21;8(2):439–45. doi: 10.1093/gbe/evw002 (PMC4779609; doi:10.1093/gbe/evw002)
Supplement: Supplementary Data [file supp_evw002_GBE_Supplementary_alignments.docx]

**Supplementary Alignment 1: Cytochrome *c* nucleic acid sequence alignment**

CLUSTAL format alignment by MAFFT (v7.182)

NT_symbB1.v1.2. ------------------------------------------------------------

HT_symbB1.v1.2. ctcaagcaatgtgaagtctatgacgtttgatgtttgcaacatgtgcattctgcatggctc

TA_symbB1.comp2 tgccagcacgagcagaatcaggaggcattgtgaaagt----------tacggtttggctc

VA379_symbB1.ES ----------------------------------------------------cttggctc

VA380_symbB1.ES ------------------------------------------------------------

NT_symbB1.v1.2. ---------------------------------------cgtgtctttggacagcggcca

HT_symbB1.v1.2. aagtcgcaaagccctggtgcagatcgaacgtgcagtccccgtgtctttggacagcggcca

TA_symbB1.comp2 aagtcgcaaagccctggtgcagatcgaacgtgcagtccccgtgtctttggacagcggcca

VA379_symbB1.ES aagtcgcaaagccctggtgcagatcgaacgtgcagtccccgtgtctttggacagcggcca

VA380_symbB1.ES ------------------------------------------------------------

NT_symbB1.v1.2. agccgtaactttcacaatgcctgtgccagagccagatgtcgaggtgccgtccggtgacac

HT_symbB1.v1.2. agccgtaactttcacaatgcctgtgccagagccagatgtcgaggtgccgtccggtgacac

TA_symbB1.comp2 agccgtaactttcacaatgcctgtgccagagccagatgtcgaggtgccgtccggtgacac

VA379_symbB1.ES agccgtaactttcacaatgcctgtgccagagccagatgtcgaggtgccgtccggtgacac

VA380_symbB1.ES ------------------------------------------------------------

NT_symbB1.v1.2. taagaagggggctaagctcttcaaggccaagtgtgctcagtgccacaccatcgagaaagg

HT_symbB1.v1.2. caagaagggggctaagctcttcaaggccaagtgtgctcagtgccacaccatcgagaaagg

TA_symbB1.comp2 caagaagggggctaagctcttcaaggccaagtgtgctcagtgccacaccatcgagaaagg

VA379_symbB1.ES caagaagggggctaagctcttcaaggccaagtgtgctcagtgccacaccatcgagaaagg

VA380_symbB1.ES -----------------tcttcaaggccaagtgtgctcagtgccacaccatcgagaaagg

*******************************************

NT_symbB1.v1.2. cggcaacgcaaagcaaggtcctccactttggggtctgattggtcgcacatccggcacatg

HT_symbB1.v1.2. cggcaacgcaaagcaaggtcctccactttggggtctgattggtcgcacatccggcacatg

TA_symbB1.comp2 cggcaacgcaaagcaaggtcctccactttggggtctgattggtcgcacatccggcacatg

VA379_symbB1.ES cggcaacgcaaagcaaggtcctccactttggggtctgattggtcgcacatccggcacatg

VA380_symbB1.ES cggcaacgcaaagcaaggtcctccactttggggtctgattggtcgcacatccggcacatg

************************************************************

NT_symbB1.v1.2. cgatggctttgcatactccgaggccaacaagaacgctgccatcgtgtggtcagacaagca

HT_symbB1.v1.2. cgatggctttgcatactccgaggccaacaagaacgctgccatcgtgtggtcagacaagca

TA_symbB1.comp2 cgatggctttgcatactccgaggccaacaagaacgctgccatcgtgtggtcagacaagca

VA379_symbB1.ES cgatggctttgcatactccgaggccaacaagaacgctgccatcgtgtggtcagacaagca

VA380_symbB1.ES cgatggctttgcatactccgaggccaacaagaacgctgccatcgtgtggtcagacaagca

************************************************************

NT_symbB1.v1.2. cttgtttgagtacttgttgaacccaaagaagtacattcctggtacaaagatggtctttgc

HT_symbB1.v1.2. cttgtttgagtacttgttgaacccaaagaagtacattcctggtacaaagatggtctttgc

TA_symbB1.comp2 cttgtttgagtacttgttgaacccaaagaagtacattcctggtacaaagatggtctttgc

VA379_symbB1.ES cttgtttgagtacttgttgaacccaaagaagtacattcctggtacaaagatggtctttgc

VA380_symbB1.ES cttgtttgagtacttgttgaacccaaagaagtacattcctggtacaaagatggtctttgc

************************************************************

NT_symbB1.v1.2. cggcatcaagaaggagaaagagcgggctgatctgattgccttcatggctgagatggctta

HT_symbB1.v1.2. cggcatcaagaaggagaaagagcgggctgatctgattgccttcatggctgagatggctta

TA_symbB1.comp2 cggcatcaagaaggagaaagagcgggctgatctgattgccttcatggctgagatggctta

VA379_symbB1.ES cggcatcaagaaggagaaagagcgggctgatctgattgccttcatggctgagatggctta

VA380_symbB1.ES cggcatcaagaaggagaaagagcgggctgatctgattgccttcatggctgagatggctta

************************************************************

NT_symbB1.v1.2. gagatcggggacaatcatgtcctggattctctttttttggaacc---cagctgctctgtt

HT_symbB1.v1.2. gagatcggggacaatcatgtcctggactctccgttttggaagccagacaggagcagagcc

TA_symbB1.comp2 gagatcggggacaatcatgtcctggactctccgttttggaagccagacaggagcagagcc

VA379_symbB1.ES gagatcggggacaatcatgtcctggattctccgttttggaagccagacaggagcagagcc

VA380_symbB1.ES gagatcggggacaatcatgtcctggattctccgttttggaagccagacaggagcagagcc

**************************.****. **** *.*.** *** ** *..

NT_symbB1.v1.2. gattcatgttgattctgtgagtaattggtactggcaaaacctgcgt-aatcacaaaggac

HT_symbB1.v1.2. agtctcatttcgttttgtgagagactcttcgctacggagggttcatccaccttcaggtac

TA_symbB1.comp2 agtctcatttcgttttgtgagagactcttcgctacggagggttcatccaccttcaggtac

VA379_symbB1.ES agtctcatttcgttttgtgagagactcttcgctacggagggttcatccaccttcaggtac

VA380_symbB1.ES agtctcatttcgttttgtgagagactcttcgctacggagggttcatccaccttcaggtac

..*.. ** .**.****** .*.* * . .*..*. * *.* *.* . *.* **

NT_symbB1.v1.2. atgggtccgcagtctgaatcatcatcaaagctcaagtgaaatagattgaaatagaacga

HT_symbB1.v1.2. agacgctctcgcgtgagtgcatcaaaa--------------------------------

TA_symbB1.comp2 agacgctctcgcgtgagtgcatcaaaaaaa-----------------------------

VA379_symbB1.ES agacgctctcgcgtgagtgcatcaaaaaaa-----------------------------

VA380_symbB1.ES agacgctctcgcgtgagtgcatcaaaaaaa-----------------------------

* . *..* *. . .. ***** *

NT: gene copy with no transcripts listed

HT: gene copy with transcripts listed

TA: Trinity assembly transcript

VA: Velvet/Oasis assembly transcripts

Kuraku S, Zmasek CM, Nishimura O, Katoh K. 2013. aLeaves facilitates on-demand exploration of metazoan gene family trees on MAFFT sequence alignment server with enhanced interactivity. Nucleic Acids Res. 41:W22-W28

**Supplementary Alignment 2: Likely chloroplast targeted ferredoxin NADP+ reductase amino acid alignment**

CLUSTAL format alignment by MAFFT (v7.245)

996.1 -----------------------------------------------MAQSVGT-ALLGA

5347.1 -----------------------------------------------MAQTLAVGALLGA

1625.1 ------------------------------------------------------------

7056.1 ---------------------------------------------------MALAFTLGA

4627.1 MVDKDFCFITFFTFATMVDSYPTVQQLPKQQLCQPSPPPTVHRINITMAQTLAVGALLGA

1066.179 MSTK-----------------------------------TENSVKMAVASAAAV-MVAGS

1066.175 ------------------------------------------------------------

303.1 ------------------------------------------------------------

996.1 AGTAFLASPGPVSRTAPSL-RGGAA--------LPGDSSSAG-MAGTAALASLAGVAMTA

5347.1 ASTAFIAPSR--SADAPAL-RGGVAQ----GAKMTGSSSVSGAMGGLAVVAAVAG-----

1625.1 ------------------------------------------------------------

7056.1 APLTAPLPIE--RSHSQTA-RFGLRD----QSSATCAASLCLGVAALVAGKRPCS-----

4627.1 ASTAFIAPSR--SADAPAL-RGGVAQ----GAKMTGSSSVSGAMGGLAVVAAVAG-----

1066.179 VSQAFVASAA--PRASPVQ-ATTWTRSGPSAGQNFGAAALT--VGAVAAVGGRAA-----

1066.175 -----MAMDL--PVARPLNLEVTRPVQAPGAAKSTSTPLVT--TSRISAVLAVAG-----

303.1 -------------MPSPTE-----------------RPTVRRCCRGLLVCTVLFW-----

996.1 AAGRRSATS----CNAAAVKKKGVKVVHGKEIPWNLFSPKAPYQGKVIENDFHPQTLTEP

5347.1 ------ATS----CRAAAVKKKGVKVVHGKEIPWNLFSPKAPYNGKVVANDFHPQTLTEP

1625.1 ------------------------------------------------------------

7056.1 ------KTS----CNAAAVKKKGVKVVHGKEIPWNLFSPKAPYKGEVVKNVVQPHTLTEQ

4627.1 ------ATS----CRAAAVKKKGVKVVHGKEIPWNLFSPKAPYNGKVVANDFHPQTLTEP

1066.179 ------LMQ----RRATAVKKKGVRVVEGKEIPWNLFSPKAPYQGSCISK----ETITSK

1066.175 ------LARCGVLRRATAVKKKGVRVVEGKEIPWNLFSPKAPYQGSCISK----ETITSK

303.1 ------VGP----SRMAAV--NGWRVVDGRSIPWNVFSPKVPFAATVVPNH------QDI

996.1 TGDANWETTHVTFDHGGKVPYIEGQSIGVIAPGPDKKGEQPAKIRLYSIASSAVGDDQSS

5347.1 TGDANWETTHVTFDHGGKVPYIEGQSIGVIAPGPDKKGEQPAKIRLYSIASSAVGDNENS

1625.1 ------------------------------------------------------------

7056.1 TGDANWETCHVTFNHAGKVPYLEGQSIGVIAPGPDKKGETPAKIRLYSIASSAVGDDETS

4627.1 TGDANWETTHVTFDHGGKVPYIEGQSIGVIAPGPTRRVSSQPRSVFIPIASSAVGDNENS

1066.179 TPLVNWETCHVIMDHGGKVPYIEGQSIGIIAPGPDKKGETPAKIRLYSIASSAPGDDETS

1066.175 TPLVNWETCHVIMDHGGKVPYIEGQSIGIIAPGPDKKGETPAKIRLYSIASSAPGDDETS

303.1 FGDGNMDYEHLTLSHHGKVPFLEGQWIGVMEPQPG----GPSKVRLYPIASSRLGDDQRG

996.1 NTVSLCVKRVVELDGK----FSNRAKGEDTADKAGTAFPDNE-VYRGVCSNHICDMTPGD

5347.1 QTVSLCVKRVVELDGK----FSNRAKGEDKADKAGTAYPDNE-VYRGVCSNHICDMSVGD

1625.1 -------------------------------------------------------MAPGD

7056.1 KTVSLCVKRVVELDGD----YANREVGEDKPDKAGTGFPENK-VYRGVCSNHICDMSPGD

4627.1 QTVFLCVKRVVELDGK----FSNRAKGEDKADKAGTAYPDNE-VYRGVCSNHICDMSVGD

1066.179 KTVSLVVKRVVEVAGKGWCEYSNVPKGKD------PEFPDAEKVYRGVCSSHICDLNAGD

1066.175 KTVSLVVKRVVEVAGKGWCEYSNVPKGKD------PEFPDAEKVYRGVCSSHICDLNAGD

303.1 KTLSLCC-------------------------TTNAGWP-------------LRQLKLGE

: *:

996.1 EVLITGPTGAEMLL---PEDPEANIIMLATGTGIAPMRSYLRLLFHDKAGAAA-------

5347.1 DVLITGPTGAEMLL---PEDPEANIIMLATGTGIAPMRSYLRLLFNDKAGAAA-------

1625.1 EVMITGPTGAEMLLPEDPEDPEANIIMLATGTGIAPMRSYLRLLFHE-AGADA-------

7056.1 EVLITGPTGAEMLL---PEDPEANIIMLATGTGIAPMRSYLRLLFNDKAGAESGLKAKFG

4627.1 DVLITGPTGAEMLL---PEDPEANIIMLATGTGIAPMRSYLRLLFNDKAGAAA-------

1066.179 DVLITGPTGAEMLL---PEDPEANMIFMATGTGIAPFRSHLRYLFHDKV-----------

1066.175 DVLITGPTGAEMLL---PEDPEANMIFMATGTGIAPFRSHLRYLFHDKV-----------

303.1 KINITGPIGTALLP---TDLREATLILVATSEGIASFRGYLRWLFHDR------------

.: **** *: :* .: **.:*::**. ***.:*.:** **::

996.1 -----DGGRKFKGLAWLFMGVPYSKSLLYDDEHQTYKKEYPDQFKYDYAVSREDK---NA

5347.1 -----DGSRKFKGLAWLFMGVPYSKSLLYDDEHQVYKKEYPDQFKYDYAVSREDK---NA

1625.1 -----DG-RKFKGLAWLFMGVPYSKFLLYDDEHQTYKKEFPSQFRYDYAVSREDK---NA

7056.1 SLLGKSAPRKFKGLAWLFMGVPYSKSLLYDDEHKEYKEKFPDNFRYDYAVSREQK---NA

4627.1 -----DGRRKFKGLAWLFMGVPYSKSLLYDDEHQVYKKEYPDQFKYDYAVSREDK-----

1066.179 ------SKGKFKGVAWMFLGVPYSQSILYDEEWKEMQAEYPDQFRYDYAVSGEEKSEKNK

1066.175 ------SKGKFKGVAWMFLGVPYSQSILYDEEWKEMQAEYPDQFRYDYAVSGEEKSEKNK

303.1 -------KERFEGLVWLLLGA----QVIYDEEFRVYQSKFPENFRFLRRSSNLDE-----

:*:*:.*:::*. ::**:* : : ::*.:*:: * ::

996.1 AGQKMYIQTKMAEYAEELWELMQDEKTHVYMCGLKGMESGMAECFGPIAEKNGLVWTEFA

5347.1 AGQKMYIQTKMAEYAEELWELMQDEKTHVYMCGLKGMESGMAECFGPIAEKNGLVWTEFA

1625.1 AGQKMYIQTKMAEYAEELWDLMQDEKTHVYMCGLKGMESGMAECFGPIAEKNGLVWTEFA

7056.1 EGQKMYIQTKMAEYAEELWDLMQDEKTHVYMCGLKGMESGMAECFGPIAEKNGKVWAEFA

4627.1 ------------------------------------------------------------

1066.179 INGEMWVQHKMMQYADDLWELVKDPKTHVYMCGLKGMESGFAECFQERVEAEGMDYAEFL

1066.175 INGEMWVQHKMMQYADDLWELVKDPKTHVYMCGLKGMESGFAECFQERVEAEGMDYAEFL

303.1 ---MLELRRSLEENCEELWKCLQLPKTYLYIIGSKDLDNIVSDVLAVV--KGPESWLDLR

996.1 KAMKKAEGHGERHGRVL

5347.1 KAMKKADRY---HVEVY

1625.1 KAM-KADRY---HVEVY

7056.1 KAMKKADRY---HVEVY

4627.1 -----------------

1066.179 KKMKKDKRY---HVEVY

1066.175 KKMKKDKRY---HVEVY

303.1 RSMQEQRRY---HCEVS

Numbers refer to scaffold number, for 1066.1, 75 and 79 refers to scaffold location.

Kuraku S, Zmasek CM, Nishimura O, Katoh K. 2013. aLeaves facilitates on-demand exploration of metazoan gene family trees on MAFFT sequence alignment server with enhanced interactivity. Nucleic Acids Res. 41:W22-W28
